# Effects of probiotic/synbiotic supplementation on body weight in patients with diabetes: a systematic review and meta-analyses of randomized-controlled trials

**DOI:** 10.1186/s12902-023-01338-x

**Published:** 2023-04-21

**Authors:** Sepideh Soltani, Marziyeh Ashoori, Fereshteh Dehghani, Fatemeh Meshkini, Zachary Stephen Clayton, Shima Abdollahi

**Affiliations:** 1 Yazd Cardiovascular Research Center, Noncommunicable Diseases Research Institute, Shahid Sadoughi University of Medical Sciences, Yazd, Iran; 2 School of Nutrition Sciences and Food Technology, Shahid Beheshti University of Medical Sciences, Tehran, Iran; 3 Department of nutritional Sciences, Texas Tech University, Lubbock, TX USA; 4 Department of Biochemistry, School of medicine, Shahid Sadoughi University of Medical Sciences, Yazd, Iran; 5 Student Research Committee, Shahid Sadoughi University of Medical Sciences, Yazd, Iran; 6 Department of Integrative Physiology, University of Colorado Boulder, Boulder, CO USA; 7 Department of Nutrition, School of Public Health, North Khorasan University of Medical Sciences, Bojnurd, Iran

**Keywords:** Obesity, Meta-analysis, Probiotics, Diabetes, Synbiotics, Systematic-review

## Abstract

**Objective:**

The aim of the present study was to assess the effect of probiotic/synbiotic supplementation on anthropometric measures in adults with diabetes, independent of body weight.

**Methods:**

PubMed, Scopus, Web of Sciences and the Cochrane Library were searched for randomized controlled trials (RCTs) up until December 14, 2022. The effect sizes were pooled using an inverse-variance random-effects model. The methodological quality of studies as well as the quality of evidence was assessed using standard tools.

**Results:**

Thirty-two RCTs met the established inclusion criteria. Overall, compared with the respective control groups, probiotic/synbiotic supplementation resulted in a significant reduction in body weight (weighted mean difference [WMD]: -0.50 kg; 95% CI: -0.83, -0.17; *I*^*2*^ = 79.8%, n = 27 studies]), body mass index (WMD: -0.24 kg/m^2^; 95% CI: -0.39, -0.09; *I*^*2*^ = 85.7%, n = 30 studies), and waist circumference (WMD: -0.90 cm; 95% CI: -1.13, -0.52; *I*^*2*^ = 0%, n = 11 studies). However, hip circumference and waist to hip ratio were not significantly improved.

**Conclusions:**

Our analysis revealed that probiotic/synbiotic supplementation may assist with weight management in patients with diabetes, especially when consumed at higher doses, in younger adults, and in participants with obesity. However, more studies are needed to elucidate the anti-obesity effects of specific strains of probiotics/synbiotics.

**Supplementary Information:**

The online version contains supplementary material available at 10.1186/s12902-023-01338-x.

## Introduction

The worldwide prevalence of obesity has tripled in the last four decades [1]. Although the origin of obesity is multifactorial, the chronic imbalance between excess energy intake and low energy expenditure is thought to be the principle cause of weight gain [2, 3]. Most individuals with overweight or obesity suffer from some form of metabolic impairment (e.g., insulin resistance), which may manifest into chronic conditions such as Type 2 diabetes mellitus (T2DM) [4]. It is well established that weight management can preserve glucose/insulin function, mitigate the progression of chronic diseases, and is recommended as a therapeutic strategy in patients with T2DM [5–9].

Recent evidence suggests that an imbalance in the gut microbiota, broadly termed “gut dysbiosis” may be associated with deregulated energy hemostasis [10]. Gut dysbiosis is also associated with obesity-related inflammation, which can exacerbate metabolic disorders in T2DM [11]. There has been a growing interest in the use of probiotic and symbiotic supplementation for modulating gut microbiota, glucose metabolism, and body weight in patients with T2DM. It has been implied that the mechanism underlying the anti-obesity effect of probiotics is mainly related to the production of short-chain fatty acids, which can in turn influence appetite-regulating hormones [12–14], improve insulin sensitivity, and increase energy expenditure [15]. Probiotics are also thought to have anti-obesity effects due, in part, to production of conjugated linoleic acid (CLA), which has shown to reduce bodyweight via heightened lipid oxidation and adipocyte apoptosis, as well as reduced lipogenesis and inflammation [16] .

Two previous systematic reviews and meta-analyses aimed to investigate the effect of probiotic supplementation on body weight in patients with T2DM [17, 18]; however, neither study reached a consensus. Body mass index (BMI) was the only anthropometric outcome included in these studies, and other metrics such as body weight, waist or hip circumference (WC or HC), waist to hip ratio (WHR), or body composition were not considered [17, 18]. In addition, there were substantial methodological limitations, such as poor search strategy, not including relevant and qualified studies, and inclusion of studies in the final analyses that used probiotics concomitantly with other interventions.

Therefore, in the present study we aimed to determine whether supplementation with probiotics and synbiotics can affect anthropometric measures in adult males and females with T2DM, using a meta-analysis approach while considering the limitations of previous studies. According to the Cochrane recommendation, we decided to include only randomized controlled trials, as they are the preferred design to draw a casual association and are least likely to be biased, especially in the context of selection bias and confounding bias which may arise in non-randomized studies [19].

## Methods

The protocol for this systematic review and meta-analysis was conducted in accordance with the Preferred Reporting Items for Systematic Reviews and Meta-Analyses (PRISMA) guidelines [20], and was registered on PROSPERO (International prospective register of systematic reviews). Registration code: CRD42021273570 Available from: https://www.crd.york.ac.uk/prospero/display_record.php?ID=CRD42021273570.

### Search strategy

A systematic literature search was performed to identify appropriate studies in four electronic databases including Scopus, PubMed, Web of Science, and the Cochrane Library until February 2021, and was updated on 14th December 2022. No language restrictions were applied. We also did not apply any key words, so as not to miss any articles. A complete electronic search strategy for PubMed is provided in Supplementary Table 1. The reference lists of the eligible studies were manually checked to identify other relevant studies. If there were missing data or we could not acquire full texts, we contacted the corresponding author.

### Inclusion criteria

Three reviewers independently evaluated the titles and abstracts of all acquired articles (SS, FM, MA). The following criteria were used to determine which studies would be included in the final analysis: (1) RCTs with either parallel or crossover design; (2) conducted in patients with pre-diabetes or T2DM; (3) compared the effects of probiotic/synbiotic supplementation or fortified foods in any strains and dosages with placebo or non-fortified foods; and (4) reported at least one of the following anthropometric measures: body weight, BMI, WC, HC, WHR or body composition.

### Exclusion criteria

We excluded trials that had a follow-up duration of less than one week, lack of placebo or control group, conducted in pregnant or lactating women, lack of access to suitable data for analysis despite contacting the corresponding author, or examined the effect of probiotics or synbiotics with adjunctive supplementation.

### Data extraction

Four reviewers (SS, FM, FD, MA) independently extracted the following data from each eligible study: study characteristics (first author’s name, publication year, study location, sample size, study design, follow up duration, study location, sample size in trial arms, type and dose of intervention/placebo, anthropometric outcomes), participants characteristics (sex, age and medical condition), as well as means and standard deviations (SDs) of anthropometric indices at baseline and at the end of the study, or mean differences (MDs) and SDs during the follow-up period. Data were cross-checked to minimize potential errors and incongruities were resolved by consensus with the corresponding author (SA). Any disagreements were resolved through consensus-based discussion. The interrater reliability was assessed and reported as Cohen’s kappa coefficient (κ) at the stage of initial and full-text screening [21].

### Risk of bias assessment and quality of evidence

The Cochrane Risk of Bias tool was used to assess methodological quality of the selected RCTs based on the following domains: *(i)* random sequence generation; *(ii)* concealment of allocation; *(iii)* blinding of participants; *(iv)* personnel and outcome assessors; *(v)* selective reporting; *(vi)* and funding bias [22]. Two authors independently assessed the domains (SS, FD) and indicated risk of bias as low, high, or unclear. Incongruities were resolved by consensus with a third author (SA).

The quality of evidence for each outcome was rated as high, moderate, low, or very low, according to the GRADE (Grading of Recommendations Assessment, Development and Evaluation) approach. The quality of evidence of a trial was first set as high and was downgraded based on the five domains referring to the risk of bias, publication bias, imprecision of results, heterogeneity, and indirectness of evidence [23].

### Statistical analysis

We examined the effect of probiotic/synbiotic supplementation or fortified foods on changes in anthropometric outcomes if there were three or more eligible studies. The mean differences (MDs) in anthropometric measures between the groups, along with their 95% confidence intervals (CIs) were used to calculate effect sizes. If a study did not provide the value for mean change, it was calculated as the difference between the final mean and the baseline mean. If standard deviations were not included for the differences between final and baseline means, values were calculated based on the formula given in Cochrane Handbook of Systematic Reviews [24]. Based on the included studies, a correlation coefficient of 0.99 was calculated for weight and 0.98 for BMI. The random-effects model (DerSimonian-Laird method) was performed to obtain the overall pooled effect size [25]. In trials with multiple arms (comparing both probiotics and synbiotics with a control group), the probiotic intervention arm was included in the main analysis, to avoid double counting of the control group in the analysis. Regarding trials use cross-over design, we included the results from paired analyses in main analysis. Regarding studies with multiple endpoints, the longer follow-up duration was considered for the final analysis. Moreover, if there were duplicate reports from the same population the most complete report was included in the final analysis.

Statistical heterogeneity between trials was tested using Cochran’s Q-test (significant level set as p < 0.1) and *I*^*2*^ (≥ 50% considered to reveal substantial heterogeneity among trials). Eight subgroup analyses were performed by age, sex, study location, study design, supplementation type (probiotics or synbiotics), probiotics/synbiotics genera (synbiotics, *Bifidobacterium*, *Lactobacillus*, *Bifidobacterium* & *Lactobacillus*, *Saccharomyces* and mixed genera), follow-up duration (< 12 wk; ≥12 wk), obesity status of participants (normal weight, overweight and obese, mixed population), probiotic dosage, and study quality (poor, fair, good) to determine potential sources of heterogeneity. We also conducted random-effects meta-regressions to assess between-group heterogeneity and examine the effect of other potential confounding variables including participants mean age, sample size, probiotic dosage, and study duration on the estimated effect size. Sensitivity analysis was employed to determine the effect of each individual study on the overall results by using the leave-one-out method. Publication bias was investigated for outcomes with ≥ 10 studies through visual inspection of the funnel plots along with statistical assessment by employing Egger’s and Begg’s tests. If there was publication bias, the trim-and-fill method was implemented to correct funnel plot asymmetry. All statistical analyses were performed using STATA version 14 (STATA Corp., College Station, Texas) with significance set at p < 0.05.

## Results

**Fig. 1:**
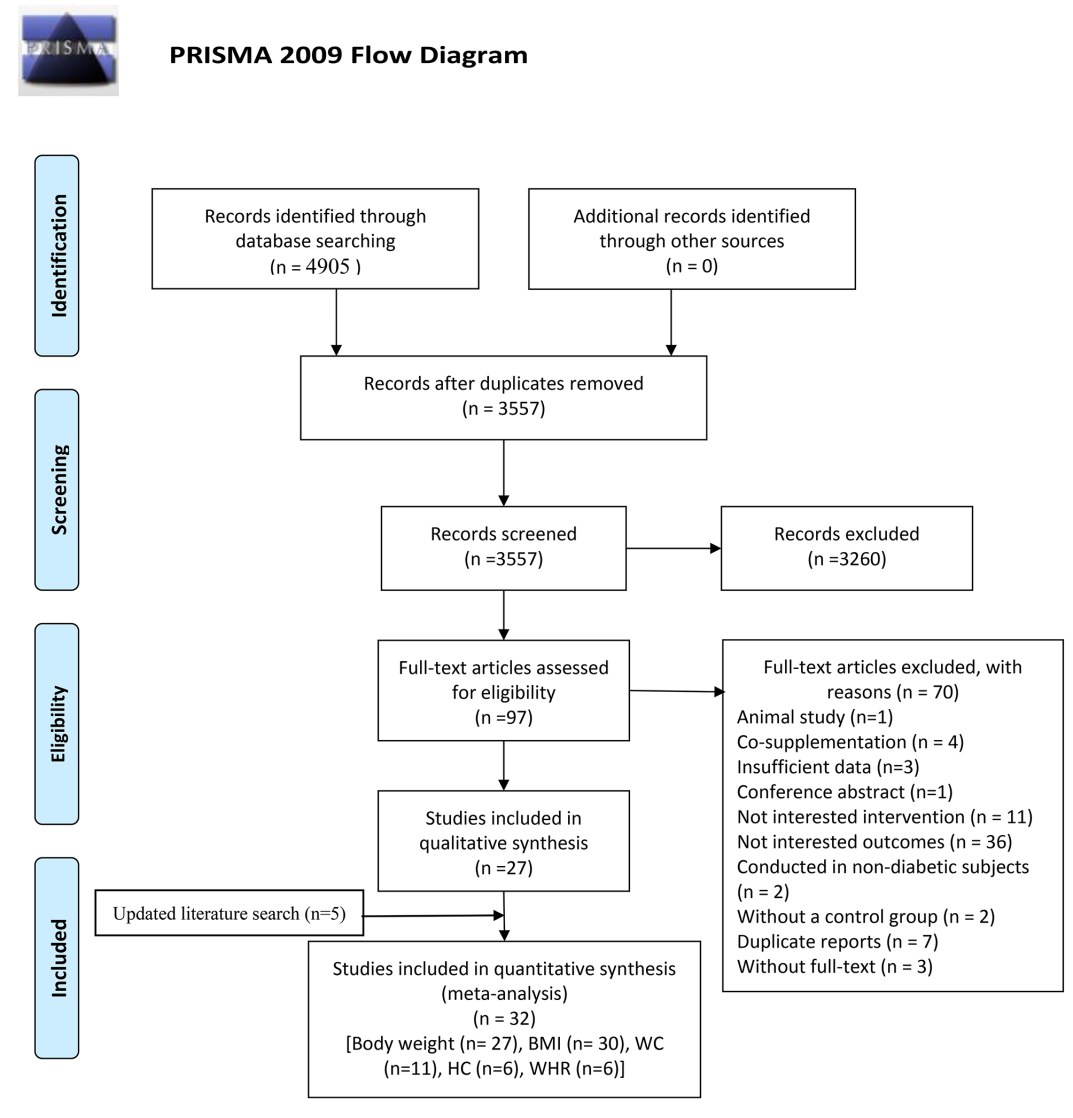
Study selection process

The primary search strategy yielded 4905 publications, of which 1348 were duplicates (Fig. 1). After screening the titles and abstracts, 97 relevant articles were selected for full-text evaluation. Seventy studies were excluded for the following reasons: animal study (n = 1), co-supplementation (n = 4), insufficient data (n = 3), conference abstract (n = 1), without interventions of interest (n = 11), without outcomes of interest (n = 36), conducted in non-diabetic subjects (n = 2), without a control group (n = 2), duplicate reports (n = 7), and without full-text (n = 3) (Supplementary Table 2). Five studies were also added after the last update [26–30]. Finally, 32 eligible RCTs were included in the meta-analysis to assess the effect of probiotic/synbiotic supplementation on body weight (n = 27) [26, 28–52], BMI (n = 30) [26–40, 42–51, 53–56], WC (n = 11) [26, 28, 30, 35–39, 44, 45, 48], HC (n = 6) [28, 30, 36, 37, 45, 48], and WHR (n = 6) [28, 36, 38, 43, 45, 55]. The reviewers’ agreement for including studies was high at the times of the abstract screening (Cohen’s kappa = 0.82) and full-text screening (Cohen’s kappa = 0.94) phases.

### Study characteristics

The characteristics of the included studies are presented in Table 1. Included trials were published from 2013 to 2020 and were conducted in Asian countries [Iran (n = 17), Japan (n = 4), Saudi Arabia (n = 2), Malaysia (n = 1), Indonesia (n = 1), Turkey (n = 1), and India (n = 1)], European countries [Denmark, Sweden, Austria and Ukraine], and one in Oceania country [New zealand]. All trials applied a parallel design, except for the study conducted by Asemi et al., which used a crossover design. The majority of the included trials recruited both sexes, whereas two trials focused exclusively on males [37] and one trial on females [28].

**Table 1:** The characteristics of trials that investigated the effect of probiotic/synbiotic supplementation on anthropometric indices in patients with diabetes and were eligible for inclusion in the meta-analysis

Author, Year/ Country	Participants, Sex/ Mean age	Study design/ Duration (weeks)	Type of diabetes/ Condition	Type of supplement	Outcomes	Results
Arani, 2019 (31)/ Iran	60, Both / 61.5	P/ 12	Type 2/ Diabetic nephropathy	Probiotic honey: B. coagulans *T11*(2.5 × 10^9^)	Weight, BMI	No significant change
Asemi, 2014 (32)/ Iran	62, Both / 50.90	C/ 6^1^	Type 2/ -	Synbiotic: L. sporogenes (2.7 10^8^) + Inulin	Weight, BMI,	No significant change
Asemi, 2013 (33)/ Iran	58, Both / 55.94	P/ 8	Type 2/ -	Probiotic: L. acidophilus (2 × 10^9^), L. casei (7 × 10^9^), L. rhamnosus (1.5 × 10^9^), L. bulgaricus (2 × 10^8^), B.breve (2 × 10^10^), B. longum (7 × 10^9^), S. thermophilus (1.5 × 10^9^)	Weight, BMI	No significant change
Barthow, 2022 (26)/ New Zealand	143, Both/ 60	P/ 26	Prediabetes/ -	Probiotic: L.rhamnosus (HN001) (6 × 10^9^ colony-forming units/day)	Weight, BMI, WC	No significant change
Firouzi, 2016 (35)/ Malaysia	101, Both / 53.82	P/ 6 &12	Type 2/ -	Probiotic: L. acidophilus *BCMC 12,130* (10^10^), L. casei *BCMC 12,313* (10^10^), L. lactis *BCMC 12,451* (10^10^), B.bifidum *BCMC 02290* (10^10^), B. Longum *BCMC 02120* (10^10^), B.infantis *BCMC 02129* (10^10^)	Weight, BMI, WC	No significant change
Horvath, 2019 (36)/ Austria	26, Both / 59.92	P/ 12 &26	Type 2/ -	Synbiotic: B. bifidum W23, B. lactis W51, B. lactis W52, L. acidophilus W37, L. casei W56, L. brevis W63, L. salivarius W24, L. lactis W58, L. lactis W19 (totally 1.5 × 10^10^)/ Galacto-oligosaccharides P11, Fructo-oligosaccharides P6 + konjac glucomannan P13 (E425), calcium carbonate (E170), zinc citrate 3-hydrate, vitamin D3 and vitamin B2 (E101)	Weight, BMI, WC, HC, WHR	Significant decrease in HC in synbiotic group after 12 weeks of intervention
Hosseinzadeh, 2013 (53)/ Iran	84, Both / 46.25	P/ 12^1^	Type 2/ -	Brewer’s Yeast (1800 mg)	BMI	No significant change
Hove, 2015 (37)/ Denmark	41, M/ 59.42	P/ 12	Type 2/ -	Fermented milk: L. helveticus Cardi04	Weight, BMI, WC, HC	No significant change
Kanazawa, 2021(27)/ Japan	86, Both/ 56	P/ 24	Type 2/ -	Synbiotic: 3.0 g dry powder containing at least 3 × 108 living L.paracasei YIT 9029 (strain Shirota:LcS) organisms, 3 × 108 living B.breve YIT 12,272 (BbrY) organisms, and 7.5 g GOS per day	BMI	No significant change
Kassaian, 2019 (54)/ Iran	86, Both / 52.97	P/ 12 &26	Prediabetes/ -	Probiotic: L. acidophilus (1.5 × 10^9^), B.bifidum (1.5 × 10^9^), B. lactis (1.5 × 10^9^), B. longum (1.5 × 10^9^) Synbiotic: Inulin + L. acidophilus (1.5 × 10^9^), B.bifidum (1.5 × 10^9^), B. lactis (1.5 × 10^9^), B. longum (1.5 × 10^9^)	BMI	No significant change
Khalili, 2019 (38)/ Iran	40, Both / 44.75	P/ 8	Type 2/ -	Probiotic: L. casei 01 (10^8^)	Weight, BMI, WC, WHR	Significant decrease in Weight, BMI, WC in probiotic group
Kobyliak, 2018 (39)/ Ukraine	53, Both / 54.28	P/ 8	Type 2/ -	Probiotic: Bifidobacterium (1 × 10^11^), Lactobacillus, Lactococcus (6 × 10^11^), Propionibacterium (3 × 10^11^)	Weight, BMI, WC	Significant decrease in WC in probiotic group compared to placebo
Kooshki, 2015 (40)/ Iran	43, Both / 54.88	P/ 8	Type 2/ -	Synbiotic: B. coagulants (1.5 × 10^7^) + Fructo-oligosaccharides	Weight, BMI	Significant decrease in weight and BMI in synbiotic group compared to placebo
Madempudi, 2019 (41)/ India	74, Both / 52.05	P/ 12^2^	Type 2/ -	Probiotic: L. salivarius UBLS22, L. casei UBLC42, L. plantarum UBLP40, L. acidophilus UBLA34, B. breve UBBr01, B. coagulans Unique IS2 (totally 6 × 10^10^)	Weight	Significant decrease in weight in probiotic group compared to placebo
Mafi, 2018 (42)/ Iran	60, Both / 59.9	P/ 12	Type 1&2/ Nephropathy	Probiotic: L. acidophilus ZT-L1 (2 × 10^9^), B.bifidum ZT-B1(2 × 10^9^), L. reuteri ZT(2 × 10^9^), L. fermentum ZT-L3(2 × 10^9^)	Weight, BMI	No significant change
Miraghajani, 2017 (43)/ Iran	40, Both / 55.25	P/ 8	Type 2/ Nephropathy	Probiotic soy milk: L. plantarum A7 (4 × 10^9^)	Weight, BMI, WHR	No significant change
Mobini, 2017(44)/ Sweden	44, Both / 65	P/ 12	Type 2/ -	Probiotic (Low & high dose): L. reuteri DSM 17,938 (10^8^) or (10^10^)	Weight, BMI, WC, Body fat	Significant increase in weight in low dose probiotic group
Mohamadshahi, 2014 (45)/ Iran	44, Both / 51	P/ 8	Type 2/ -	Probiotic yogurt: B.animalis subsp. lactis Bb12 (DSM 10,140), L. acidophilus La5 (Totally 11.1 × 10^8^)	Weight, BMI, WC, HC, WHR, Body fat	No significant change
Mohseni, 2018 (46)/ Iran	60, Both / 60.55	P/ 12	Type 1&2/ Diabetic foot ulcer	Probiotic: L. acidophilus (2 × 10^9^), L. casei (2 × 10^9^), L. Fermentum (2 × 10^9^), B. bifidum (2 × 10^9^)	Weight, BMI	No significant change
Naito, 2017 (47)/ Japan	98, Both / 47.01	P/ 4& 8	Prediabetes/ -	Fermented milk: L. casei Shirota YIT 9029 (1 × 10^11^)	Weight, BMI, Body fat	No significant change
Razmpoosh, 2018 (48)/ Iran	60, Both / 59.95	P/ 6^2^	Type 2/ -	Probiotics: L. acidophilus (2 × 10^9^), L. casei (7 × 10^9^), L. rhamnosus (1.5 × 10^9^), L. bulgaricus (2 × 10^8^), B.breve (3 × 10^10^), B.longum (7 × 10^9^), S. thermophiles (1.5 × 10^9^)	Weight, BMI, WC, HC	No significant change
Rustanti, 2022(28)/ Indonesia	36, F/ 44.11	P/ 11	Type 2/ -	Skim milk powder containing L.plantarum Dad-13 (1010 CFU)	Weight, BMI, WC, HC, WHR	No significant change
Sabico, 2017 (52)/ Saudi Arabia	78, Both / 47.3	P/ 12^2^	Type 2/ -	Probiotics: B.bifidum W23, B.lactis W52, L. acidophilus W37, L. brevis W63, L. casei W56, L. salivarius W24, L^1^. lactis W19, La. lactis W58 (totally 5 × 10^9^)	Weight, BMI, WHR	Significant decrease in WHR in probiotic group compared to placebo
Sabico, 2018 (55)/ Saudi Arabia	61, Both / 47.3	P/ 12 & 26^2^	Type 2/ -	Probiotics: B.bifidum W23, B. lactis W52, L. acidophilus W37, L. brevis W63, L. casei W56, L. salivarius W24, L^1^. lactis W19, La. lactis W58 (totally 5 × 10^9^)	BMI, WHR	No significant change
Sahin, 2022 (29)/ Turkey	126, Both/ 50.8	P/ 4	Prediabetes and Type 2/ -	Probiotics: B. animalis subsp.lactis (BB-12)(4.6 mg)	Weigh, BMI	No significant change
Sato, 2017 (56)/ Japan	68, Both / 64.5	P/ 8 &16	Type 2/ -	Fermented milk: L. casei Shirota (4 × 10^10^)	BMI	No significant change
Soleimani, 2016 (49)/ Iran	60, Both / 56.7	P/ 12	Type 1&2/ Hemodialysis	Probiotic: L. Acidophilus (2 × 10^9^), L. casei (2 × 10^9^), B.bifidum (2 × 10^9^)	Weight, BMI	No significant change
Tajabadi-Ebrahimi, 2014 (50)/ Iran	81, Both / 52.35	P/ 8^1^	Type 2/ -	Probiotic bread: L. sporogenes (1 × 10^8^) Synbiotic bread: / L. sporogenes (1.2 × 10^8^) + Inulin	Weight, BMI	No significant change
Tajabadi-Ebrahimi, 2017 (34)/ Iran	60, Both / 64.1	P/ 12	Type 2/ coronary heart disease	Synbiotic: L. acidophilus (2 × 10^9^), L. casei (2 × 10^9^), B.bifidum (2 × 10^9^) + Inulin	Weight, BMI	No significant change
Toshimitsu, 2020 (51)/ Japan	126, Both/ 50.91	P/ 12	Pre-diabetes/ -	Probiotic yogurts: L. plantarum OLL2712 (5 × 10^9^)	Weight, BMI	No significant change
Velayati, 2021(30)/ Iran	43, Both/ 60.31	P/ 12	Type 2/ -	Probiotic: B. Coagulans GanedenBC30 (2 × 10^11^), L.rhamnosus GG (2 × 10^10^), L. acidophilus and 500 mg fructo oligosaccharide and 0.7% Natural Orange flavor	Weight, BMI, Waist, HC	No significant change

^1^Three times a day

^2^Twice-a-day

A, Acetobacter; B. Bifidobacterium; Ba. Bacillus; BMI, body mass index; C, crossover; HC, hip circumference; L. Lactobacillus; La. Lactococcus; P, Parallel; Pr. Propionibacterium; WC, waist circumference; WHR, waist to hip ratio

The majority of the included studies enrolled patients with T2DM (n = 25), while the remaining were conducted in patients with pre-diabetes (n = 4), and a mix population of both Type 1 and 2 diabetes (n = 3). Treatment duration ranged from 4 to 26 weeks. Different genus of microbial organisms was assessed across studies including *Lactobacillus* (10 trials), *Bifidobacterium* (n = 1), *Bacillus* (one trial), a mixture of *Lactobacillus* & *Bifidobacterium* (nine trials), *Saccharomyces* (one trial), and multi-genus mixtures (three trials). The remaining six trials evaluated the effect of synbiotics on anthropometric measures. Among the included studies, the daily dose of probiotic supplementation ranged from 1 × 10^8^ to 3 × 10^11^ colony forming units (CFU). The effects of fortified-foods including fermented milk (n = 4), probiotic yogurt, probiotic honey, probiotic soy milk, and probiotic/synbiotic bread were also assessed in the included studies.

A number of side-effects were reported following supplementation including gastric disturbances, [35, 39, 44, 54] flatulence, [36, 41, 55] nausea, [39] hypoglycemia, [44] headache, [44] diarrhea [36, 37, 39] and constipation [41].

### Risk of bias and quality of evidence

The methodological quality of eligible studies was assessed using Cochrane Collaboration’s tool (Supplementary Table 3). Twenty-three studies were rated as good quality, five as fair quality, and four were judged as having poor methodological quality. The main source of bias was the lack of explanation regarding concealment procedures. Although most of the included studies provided a description of participant blinding and outcome assessments, except for two studies [27, 56] which rated as high risk of bias for blinding, there was no risk of bias for selective reporting, or funding sources.

The quality of evidence was very low for the effect of probiotic/synbiotic supplementation on body weight, low for BMI, HC and WHR and moderate for WC **(**Supplementary Table 4).

### Meta-analysis

**Fig. 2:**
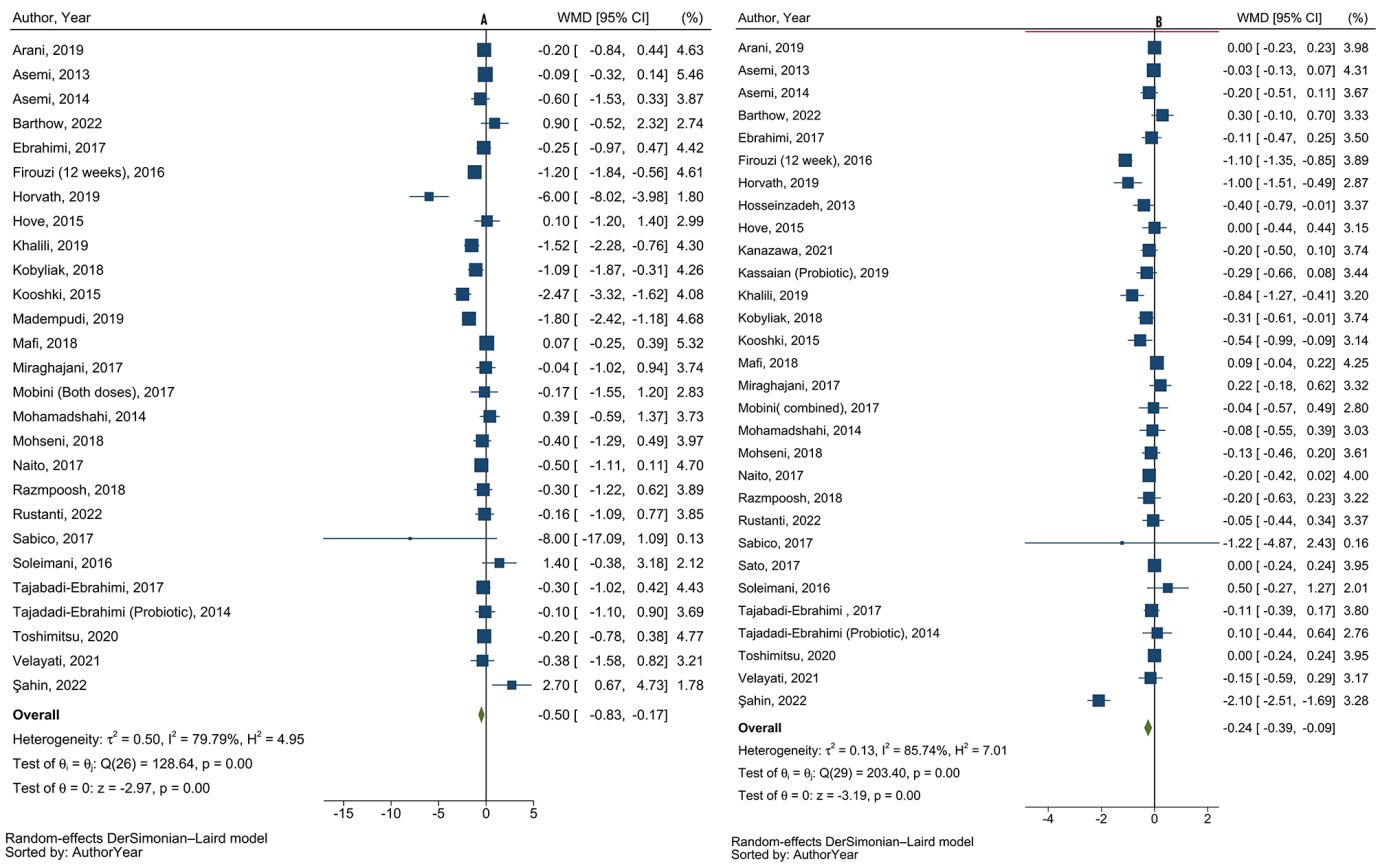
Forest plot of randomized controlled clinical trials illustrating weighted mean difference (WMD) in **A**; body weigh change (kg), **B**: BMI change (kg/m^2^) between the probiotics/synbiotics supplementation and control groups for all eligible studies Analysis was conducted using random effects model

***Weight.***The meta-analysis of 27 trials (n = 1787 participants) indicated that probiotic/synbiotic supplementation resulted in significant weight loss compared with placebo, with a considerable level of heterogeneity (WMD = -0.50 kg; 95% CI: -0.83, -0.17; P = 0.003; *I*^*2*^ = 79.8%; P-heterogeneity < 0.001) (Table 2; Fig. 2 (A)). Subgroup analysis according to the study quality and age could reduce the heterogeneity. Subgroup analyses revealed a greater weight loss following probiotic/synbiotic supplementation in: *(i)* studies conducted in Asia; *(ii)* participants under 60 years of age; *(iii)* participants with overweight and obesity; *(iv)* studies using synbiotic supplementation; *(v)* studies with a higher dose of probiotics; and *(vi)* studies with good methodological quality. A greater weight loss was also observed when probiotic/ synbiotic supplementation was administered for greater than 12 weeks (Supplementary Table 5).

**Table 2:** Meta-analysis showing the effect of probiotics/synbiotics supplementation on anthropometric indices in patients with diabetes using random effects model

		Meta-analysis		Heterogeneity		
Outcomes	Number of Studies	WMD (95%CI)	P effect	Q statistic	P within group	I^2^ (%) (95%CI)
Weight (kg)	27	-0.50 (-0.83, -0.17)	0.003	128.64	< 0.001	79.8 (71, 86)
BMI (kg/m^2^)	30	-0.24 (-0.39, -0.09)	0.001	203.40	< 0.001	85.7 (81, 89)
WC (cm)	11	-0.90 (-1.13, -0.52)	< 0.001	3.13	0.99	0 (0, 60)
HC (cm)	6	-1.34 (-2.80, 0.12)	0.07	2.76	0.74	0 (0, 75)
WHR	6	-0.01 (-0.04, 0.02)	0.36	15.08	0.01	66.8 (21, 86)

BMI, body mass index; HC, hip circumference; WC, waist circumference; WHR, waist to hip ratio

***BMI.*** The overall meta-analysis of 30 trials (n = 2098 participants) showed a significant reduction in BMI following probiotic/synbiotic supplementation in patients with diabetes (WMD= -0.24 kg/m^2^; 95% CI: -0.39, -0.09; P = 0.001; *I*^*2*^ = 85.7%; P-heterogeneity < 0.001) (Table 2; Fig. 2 (B)). A significant between-study heterogeneity was also detected, which appeared to be associated with different types of probiotic supplementation, age, and BMI status. The subgroup analysis revealed that supplementation with probiotics/synbiotics resulted in a significant reduction in BMI when participants: *(i)* were less than 60 years of age; *(ii)* lived in Asia; *(iii)* were overweight and obese; *vi)* when the study was rated as having good quality; and *v)* were followed for less than 12 weeks. Furthermore, supplementation with synbiotics or a mixed probiotic product showed a significant reduction in BMI (Supplementary Table 6).

**Fig. 3:**
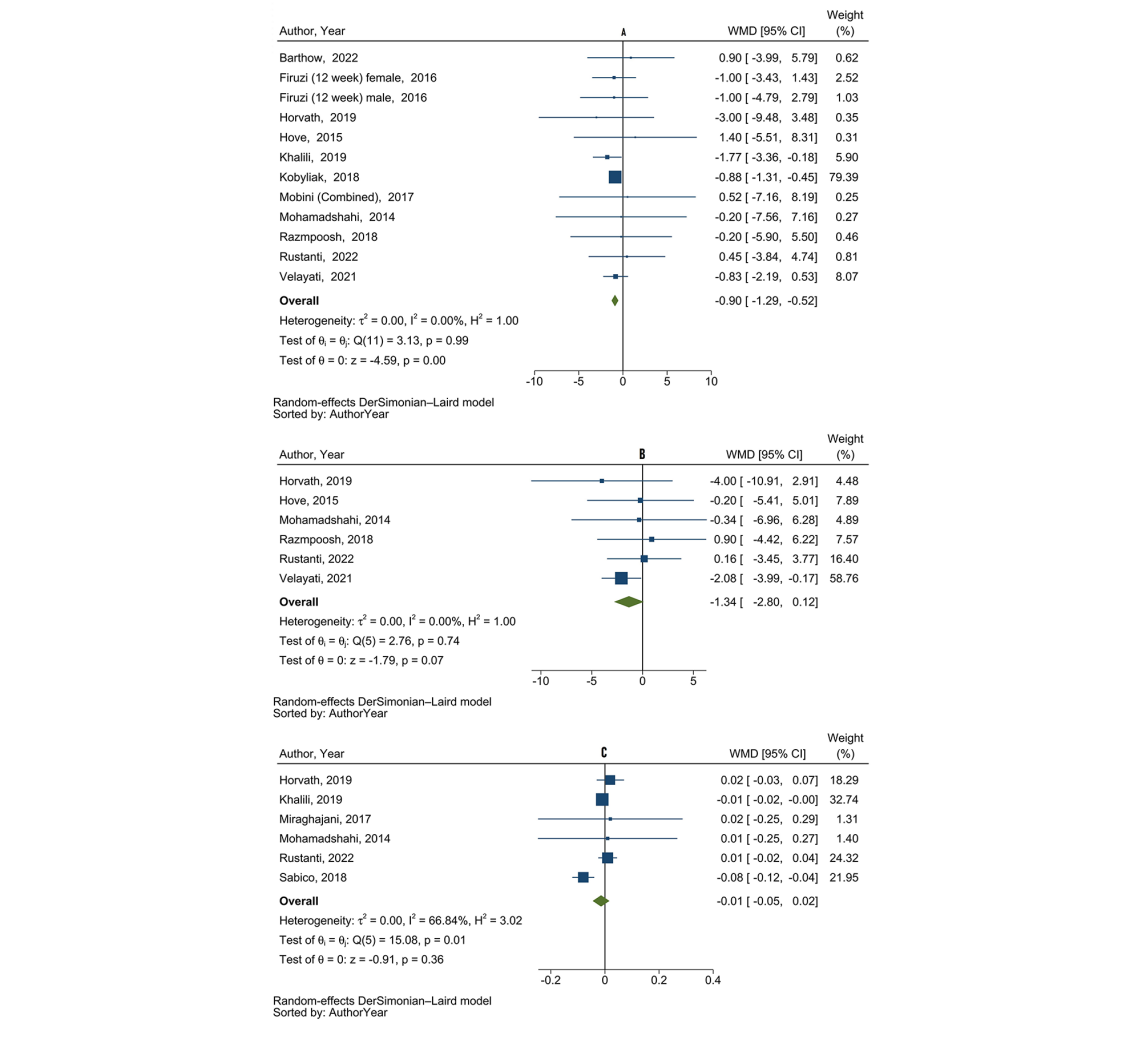
Forest plot of randomized controlled clinical trials illustrating weighted mean difference (WMD) in **A**: waist circumference change (cm), **B**: hip circumference change (cm), and **C**: waist to hip ratio change between the probiotics/synbiotics supplementation and control groups for all eligible studies. Analysis was conducted using random effects model

***WC.*** The overall estimate of the 11 studies (n = 631 participants) showed a significant reduction in WC following probiotic/synbiotic supplementation in patients with diabetes, with no evidence of between-study heterogeneity (WMD= -0.90 cm; 95% CI: -1.13, -0.52; P < 0.001; *I*^*2*^ = 0%; P-heterogeneity = 0.99) (Table 2; Fig. 3 (A)). Subgroup analysis revealed a significant decrease in WC following probiotic/synbiotic supplementation in studies: *(i)* conducted in participants under 60 years of age; *(ii)* in which participants were overweight and obese; and *(iii)* with good quality that were less than 12 weeks. In addition, Asians demonstrated a greater decrease in WC following supplementation with probiotics/synbiotics compared with Europeans. We also found a significant decrease in WC following supplementation with a higher dose of probiotics (> 10 × 10^9^ CFU/day) (Supplementary Table 7).

***HC.*** Pooled data from six trials (n = 250 participants) showed that supplementation with probiotics/synbiotics had no significant effect on HC, with no evidence of heterogeneity (WMD= -1.34 cm; 95% CI: -2.80, 0.12 cm; P = 0.07; *I*^*2*^ = 0.0%; P-heterogeneity = 0.74) (Table 2; Fig. 3 (B)). Subgroup analysis was not performed due to insufficient data.

***WHR.*** Pooled data from six trials (n = 247 participants) demonstrated no significant effect of probiotics/synbiotics supplementation on WHR, with a substantial between-study heterogeneity (WMD= -0.01; 95% CI: −0.04, 0.02; P = 0.36; *I*^*2*^ = 66.8%; P-heterogeneity = 0.01) (Table 2; Fig. 3 (C)). However, due to the low number of studies we were unable to conduct subgroup analyses, and the source of heterogeneity remained undetermined.

***Body fat mass.*** The effect of probiotic/synbiotic supplementation on body fat mass was investigated across three trials [44, 45, 47]. Two trials reported change in body fat mass as percent change and one trial as absolute change (kg) [44, 47]. No significant effect of probiotic/synbiotic supplementation on body fat mass was reported [45].

### Meta-regression

Meta-regression showed that mean age, sample size and probiotic dosage did not influence the estimated effect size of any anthropometric measure. However, the effect of probiotic/synbiotic supplementation on body weight (Slope: 0.03, CI: 0.00, 0.05; P = 0.02) was associated with the number of participants (Supplementary Table 8).

### Sensitivity analysis and publication bias

The leave-one-out sensitivity analysis was performed for each outcome, and did not influence the results. Publication bias was examined for body weight and BMI, and visual inspection of funnel plots suggested symmetry estimate, which was confirmed by related tests (Begg’s test, P = 0.60; Egger’s test, P = 0.63 for body weight; Begg’s test, P = 0.13; Egger’s test, P = 0.13 for BMI, for waist circumference; Begg’s test, P = 0.53; Egger’s test, P = 0.67) (Supplementary Fig. 1).

## Discussion

The results of this meta-analysis demonstrate that supplementation with probiotics/synbiotics may result in improvements in body weight, WC, and BMI in patients with diabetes. We also found that probiotic/synbiotic supplementation produces potentially greater anti-obesity effects when administered at higher doses in younger adults and in individuals living in Asian countries. In addition, probiotic/synbiotic supplementation is effective in reducing body weight in patients with obesity, but not in normal-weight individuals. Moreover, it appears that synbiotics may exert greater weight loss than probiotics.

Although, some of the previous meta-analyses suggest that there is no effect of probiotics on body weight in patients with diabetic nephropathy [57], women with polycystic ovary syndrome [58], overweight and obese participants [59], or adults with metabolic syndrome [60], other studies indicate an improvement in anthropometric measures following probiotic supplementation in the general population [61] or in overweight and obese subjects [62–64]. There are two published meta-analyses examining the effect of probiotics on anthropometric measures in people with Type 2 diabetes, both did not observe a significant effect on BMI [17, 18]. However, in these meta-analyses, BMI is the only reported anthropometric measure, and as previously explained, these studies have several methodological limitations which may have affected the results.

There is currently debate regarding whether the observed weight loss elicited by probiotics is clinically significant. Consistent with previous meta-analyses [61, 62, 64] we observed a relatively small reduction in body weight following probiotic/synbiotic supplementation. Although a 5% reduction in body weight is accepted as a meaningful weight loss, there is some evidence to suggest that a lower percentage of weight loss may be beneficial [65, 66]. It appears that probiotics alone may not be clinically effective for weight loss, but probiotic supplementation could be introduced as a safe complementary approach to common weight-loss strategies (i.e., calorie restriction and increased physical activity). Along with the weight-reducing effects of probiotics, these supplements have favorable safety profiles and positively influence metabolic function in patients with diabetes [66]. As such, the addition of probiotic supplements to a standard diabetes treatment regimen may be advantageous.

The exact mechanisms behind the anti-obesity effects of probiotics are not completely understood; however, some mechanisms have been proposed. Probiotics have shown to affect bile acid metabolism in the intestine [67], which in turn can lead to decreased fat absorption and increased lipolysis. Moreover, gut microbiota may influence: (i) fatty acid oxidation and lipogenesis (via activation of AMPK) [68]; (ii) appetite (by the production of short chain fatty acids [SCFAs] and their signaling function which affects peptide YY, glucagon-like peptide 1, and ghrelin) [69]; (iii) triglyceride accumulation in adipose tissue (through intestinal fasting induced adipose factor and its regulatory role on lipoprotein lipase [69]; and iv) by producing SCFAs that may enhance insulin signaling, apoptosis of adipocytes, and attenuate lipid accumulation [70]. Although probiotics could influence gut microbiota abundance and composition to improve metabolic health, all of the mentioned metabolic effects could strictly be due to weight loss following probiotic supplementation; however, this needs to be further investigated.

A significant improvement in anthropometric measures (body weight, BMI, and WC) was observed when analysis was restricted to studies conducted among overweight and obese patients and not normal weight individuals or mixed populations, perhaps due to higher starting baseline body weight which provided greater opportunity to see changes. Also, gut microbiota composition has shown to be altered with obesity [71], and modulation of intestinal microbiota with probiotic supplementation could explain the greater weight loss observed in obese subjects. SCFAs are also produced as the final products of soluble fiber fermentation. This could explain our finding regarding the greater weight loss observed following synbiotic supplementation. Moreover, the anti-obesity effects of dietary fiber have been well established [72].

We observed higher weight reduction in Asian relative to European countries. A previous meta-analysis also showed that the favorable metabolic effects of probiotics became non-significant when RCTs conducted in Iran were excluded [61]. Multiple factors such as genetics, diet, lifestyle, and other environmental factors may affect the response to probiotics. For instance, higher consumption of fermentable carbohydrates in Asian countries [73] (which provide a supportive environment for probiotic function) could be a dietary factor affecting the results. However, it should be noted that in the present meta-analysis, the number of trials conducted in Asian countries were substantially greater than those conducted in European countries (23 vs. 4, respectively).

In line with previous meta-analyses [61, 63, 64, 74], our results suggest that in addition to the type of probiotics, dose of supplementation also plays an important role in promoting the anti-obesity effects of probiotics. For example, high dose supplementation (> 10 × 10^9^ CFU/day) resulted in a significant weight reduction, while lower doses did not. However, one meta-analysis reported greater weight reduction following medium and low dose probiotic supplementation [63]. The different cutoffs used to categorize probiotic dosage may explain the inconsistency, as John et al. [63] defined medium dosage as 1–30 × 10^9^ CFU/day, which overlaps with both low and high dose categories in the present study. Further research is needed to identify the maximally tolerated dose for weight reduction and/or the minimal effective dose for promoting weight loss.

Our results also showed probiotic/synbiotic supplementation is more effective in younger participants (< 60 yrs). It has been reported that there is an age-dependent variation in the composition of gut microbiota and its related metabolic pathways [75, 76], which may lead to an altered response to probiotics supplementation. However, the limited number of trials in older participants makes it difficult to reach a finite conclusion.

The present meta-analysis pooled data from RCTs to draw a causal association between probiotic/synbiotic supplementation and body weight measures in patients with diabetes. In addition, using a comprehensive search strategy, without any restriction on outcomes ensured that we obtained all eligible studies. The methodological quality of the studies was assessed and showed good quality for most of the included studies. However, there are some limitations that should be considered while interpreting these findings. More than half of the included RCTs were conducted in Iran and only four studies (out of 27 included trials) were conducted among non-Asian populations (i.e., in European countries), which limits the generalizability of our results. Furthermore, some characteristics including baseline weight status, duration of diabetes, change in energy intake and physical activity, dietary fiber intake, and the medications used were not controlled for in the included trials. Although the statistical heterogeneity was relatively high for body weight, BMI, and WHR, the sources of heterogeneity were identified through subgroup analyses. For BMI and body weight, factors including age of participants, methodological quality of RCTs, and baseline weight status could explain the statistical heterogeneity; however, subgroup analysis for WHR was not performed (due to the low number of studies) and source of heterogeneity remained unclear. We conducted a subgroup analysis based on the probiotic genus; however, after dividing the groups, the number of studies was not adequate to assess the effect of specific probiotic strains. Finally, it was not clear whether probiotic/synbiotic supplementation could modify gut microbiota composition, and as such, this variable should be assessed in future studies.

## Conclusion

In conclusion, although the anti-obesity effect of probiotics/synbiotics is not clinically significant, it may play a facilitating role in weight reduction in patients with obesity and diabetes, and could be used as a complementary therapeutic approach. However, before probiotics/synbiotics can be widely recommended, it is necessary to evaluate the optimal dose and strain.

## Electronic supplementary material

Below is the link to the electronic supplementary material.

Supplementary Material 1

## Data Availability

The datasets used and/or analyzed during the current study are available from the corresponding author on reasonable request.
