# Supplementary material for: Effects of probiotic/synbiotic supplementation on body weight in patients with diabetes: a systematic review and meta-analyses of randomized-controlled trials
Source: BMC Endocr Disord. 2023 Apr 21;23:86. doi: 10.1186/s12902-023-01338-x (PMC10120130; doi:10.1186/s12902-023-01338-x)
Supplement: Supplementary file 1 — Supplementary Material 1 [file 12902_2023_1338_MOESM1_ESM.docx]

**Supplementary Table 1.** Search strategies used for online databases

The following databases were searched:

- PubMed
- Scopus
- Web of Science
- Cochrane Library

The search strategy was to combine searches of:

| Probiotic | Probiotics [MeSH Terms] OR probiotic*[tiab] OR "cultured milk products"[ MeSH Terms] OR "milk product"[tiab] OR yogurt[tiab] OR kefir[tiab] OR “dairy product”[tiab] OR Bifidobacterium[MeSH Terms] OR Bifido*[tiab] OR Bacteroides[MeSH Terms] OR Bacteroides[tiab] OR Lactobacillus[MeSH Terms] OR Lactobacil*[tiab] OR Lactobacillaceae[MeSH Terms] OR Pediococcus[MeSH Terms] OR Pediococcus[tiab] OR “Fermented Foods and Beverages”[MeSH Terms] OR “Fermented milk”[tiab] OR Nissle[tiab] OR “Fermented Foods”[tiab] OR Streptococ*[tiab] OR Saccharomyces[MeSH Terms] OR Saccharomy*[tiab] OR Enterococcus[MeSH Terms] OR Enterococcus[tiab] OR Lactobacillales [MeSH Terms] OR “Lactic acid bacteria”[tiab] OR “Bacillus mesentericus”[tiab] OR “Escherichia coli”[MeSH Terms] OR “Escherichia coli”[tiab] OR acidophilus[tiab] OR microorganism∗[tiab] OR buttermilk[tiab] OR lassi[tiab] OR doogh[tiab] OR dough[tiab] OR dahi[tiab] OR amasi[tiab] OR filmjolk[tiab] OR chal[tiab] OR “VSL#3”[tiab] OR Synbiotics[MeSH Terms] OR Symbiotic*[tiab] OR Synbiotic*[tiab] |
| --- | --- |
| Population | Diabetes Mellitus[mesh terms] OR “Diabetes Mellitus” [tiab]OR hyperglycemia [tiab]OR diabet*[tiab] |
| Study design | "Randomized"[Title/Abstract] OR "random"[Title/Abstract] OR "random allocation"[Title/Abstract] OR "Random assignment"[Title/Abstract] OR "Intervention"[Title/Abstract] OR "Clinical trial"[Title/Abstract] OR "Randomized controlled trial"[Title/Abstract] OR "Randomized controlled trials"[Title/Abstract] OR "trial"[Title/Abstract] OR "Placebo"[Title/Abstract] OR "Double-blind"[Title/Abstract] OR "Single-blind"[Title/Abstract] OR "clinical trials as topic"[MeSH Terms] OR "random allocation"[MeSH Terms] OR "Randomised"[Title/Abstract] OR "Randomised clinical trials"[Title/Abstract] OR "Randomised clinical trial"[Title/Abstract] OR "RCT"[Title/Abstract] |

“Probiotic, “population”, and “study design” related terms

**Supplementary Table 2**. References for excluded studies

| Animal study (n=1) (1) |
| --- |
| Co-supplementation (n = 4) (2-5) |
| Insufficient data (n=3) (6-8) |
| Meeting abstract (n=1) (9) |
| Without interested intervention (n = 11) (10-20) |
| Without interested outcomes (n = 36) (21-56) |
| Conducted in non-diabetic subjects (n = 2) (57, 58) |
| Without a control group (n = 2) (59, 60) |
| Duplicate reports (n = 7) (61-67) |
| Without full-text (n = 3) (68-70) |

| Supplementary Table 3. Study quality and risk of bias assessment using the Cochrane Collaboration’s tool. | | | | | | | | |
| --- | --- | --- | --- | --- | --- | --- | --- | --- |
| First author, year | **Sequence generation** | **Allocation concealment** | **Blinding of participants** | **Blinding of outcome assessment** | **Incomplete outcome data** | **Selective reporting** | **Funding** | **Overall** |
| Arani, 2019 | L | U | L | L | L | L | L | Fair |
| Asemi, 2013 | L | L | L | L | L | L | L | Good |
| Asemi, 2014 | L | L | L | L | L | L | L | Good |
| Barthow, 2022 | L | L | L | L | L | L | L | Good |
| Ebrahimi, 2017 | L | L | L | L | L | L | L | Good |
| Firouzi, 2016 | L | L | L | L | L | L | L | Good |
| Horvath, 2019 | L | L | L | L | L | L | L | Good |
| Hosseinzadeh, 2013 | L | U | L | L | L | L | L | Fair |
| Hove, 2015 | L | L | L | L | L | L | L | Good |
| Kanazawa, 2021 | L | U | H | H | H | L | L | Poor |
| Kassaian, 2019 | L | L | L | L | L | L | L | Good |
| Khalili, 2019 | L | L | L | L | L | L | L | Good |
| Kobyliak, 2018 | L | L | L | L | L | L | L | Good |
| Kooshki, 2017 | U | U | L | L | L | L | L | Poor |
| Madempudi, 2019 | L | L | L | L | L | L | L | Good |
| Mafi, 2018 | L | L | L | L | L | L | L | Good |
| Miraghajani, 2017 | L | L | L | L | L | L | L | Good |
| Mobini, 2017 | U | U | L | L | L | L | L | Fair |
| Mohamadshahi, 2014 | L | U | L | L | L | L | L | Fair |
| Mohseni, 2018 | L | L | L | L | L | L | L | Good |
| Naito, 2017 | L | L | L | L | L | L | L | Good |
| Razmpoosh, 2018 | L | L | L | L | L | L | L | Good |
| Rustanti, 2022 | L | U | L | L | L | L | L | Fair |
| Sabico, 2017 | L | L | L | L | L | L | L | Good |
| Sabico, 2018 | L | L | L | L | L | L | L | Good |
| Sahin, 2022 | L | U | U | U | H | L | L | Poor |
| Sato, 2017 | L | L | H | H | L | L | L | Poor |
| Soleimani, 2016 | L | L | L | L | L | L | L | Good |
| Tajabadi-Ebrahimi, 2014 | L | L | L | L | L | L | L | Good |
| Tajabadi-Ebrahimi, 2016 | L | L | L | L | L | L | L | Good |
| Toshimitsu, 2020 | L | L | L | L | L | L | L | Good |
| Velayati, 2021 | L | L | L | L | L | L | L | Good |

| **Supplementary Table 4**. GRADE assessment of confidence in estimates of effect in randomized trials | | | | | | | |
| --- | --- | --- | --- | --- | --- | --- | --- |
| Outcome | Participants (studies) | Risk of bias^1^ | Inconsistency^2^ | Indirectness^3^ | Imprecision^4^ | Publication bias^5^ | Certainty of evidence^6^ |
| Weight | 1439 (23) | Not Serious ^7^ | Serious (I^2^=81.1)^8^ | Serious^9^ | Serious^10^ | Not Serious ^11^ | Very Low^12^ |
| BMI | 1664 (25) | Not Serious ^13^ | Serious (I^2^=78.4)^14^ | Serious^15^ | Serious^16^ | Not Serious ^17^ | Low^18^ |
| Waist | 409 (8) | Not Serious ^19^ | Not Serious (I^2^=0) | Not Serious | Serious^20^ | Not Assessed^21^ | Moderate^22^ |
| HC | 171 (4) | Not Serious ^23^ | Not Serious (I^2^=0) | Serious^24^ | Serious^25^ | Not Assessed^26^ | Low^27^ |
| WHR | 211 (5) | Not Serious ^28^ | Serious (I^2^=70.2)^29^ | Serious^30^ | Serious^31^ | Not Assessed^32^ | Low^33^ |

1. Cochrane scale was used to assess trial quality across the following domains: random sequence generation, allocation concealment, blinding of participants and personnel, blinding of outcome assessment, incomplete outcome data, and selective outcome reporting. Trials were considered as good quality if all domains were low RoB, fair quality if one domain was high RoB or two domains were unclear RoB, and poor quality if two or more domains were high or unclear RoB. We downgraded one level (Serious) for RoB if more than 50% of RCTs were at high RoB while the direction and significance of effect sizes were different between overall analysis vs high quality trials category.
2. We downgraded one level for inconsistency when I^2^ was more than 50% (Serious). Where I^2^>50%, and predefined subgroups (calorie restriction, behavioral, physical activity) were not explain the source of heterogeneity, we performed sensitivity analyses by excluding one or two trials that explained most of the heterogeneity among included trials. In such cases, if values of I^2^ became <50% and the direction and significance did not change, we did not downgrade for inconsistency. The I^2^ was ≤ 50% inconsistency considered as Not serious limitation
3. We downgraded one level for indirectness if more than 75% included trials have been conducted in the same geographical location. We also downgraded one level for indirectness if the number of trial for each intervention was less or equal to 5 (low sample size limits the generalizability of the findings).
4. We downgraded one level for imprecision if the number of participants was less than 400. In case of more than 400 participants were enrolled for the interested intervention, we downgraded one level if point estimate was smaller than 5% baseline weight (4.50 kg), or point estimate surpassed 4.50 kg, and 95%CI overlapped 1kg.
5. We assessed for potential publication bias when the number of included trials for each the interested intervention were ≥10. We downgraded if there was evidence of bias with Egger's test (P<0.05) or there was evidence of asymmetry in the funnel plot.
6. The overall certainty of evidence.
7. The RoB for 4 of 23 trials were fair and poor quality.
8. I^2^=81.1, P heterogeneity <0.001. The predefined subgroups and sensitivity analysis did not explain the source of heterogeneity. Downgraded
9. More than the half of trials (14 of 23) were conducted in Iran that limited the generalizability of the findings. Downgraded.
10. More than 400 participants were available. The point estimate was smaller than 5% baseline weight (WMD=-0.62kg). Downgraded.
11. There was no evidence of asymmetry in the funnel plot and the results from the Egger’s test (p=0.09) and Begg’s test (p=0.75) showed no evidence of publication bias.
12. Data from RCTs begin with a grade of “HIGH”. Downgraded for inconsistency, indirectness and imprecision.
13. The RoB for 6 of 25 trials were fair and poor quality.
14. I^2^=78.4, P heterogeneity <0.001. The predefined subgroups and sensitivity analysis did not explain the source of heterogeneity. Downgraded
15. More than the half of trials (16 of 25) were conducted in Iran that limited the generalizability of the findings. Downgraded.
16. More than 400 participants were available. The point estimate was smaller than minimal clinical significance (WMD=-0.20 kg/m^2^). Downgraded.
17. There was no evidence of asymmetry in the funnel plot and the results from the Egger’s test (p=0.133) and Begg’s test (p=0.134) showed no evidence of publication bias.
18. Data from RCTs begin with a grade of “HIGH”. Downgraded for RoB, inconsistency, indirectness and imprecision.
19. The RoB for 2 of 8 trials were fair quality.
20. More than 400 participants were available. The point estimate was smaller than minimal clinical significance (WMD=-0.93 cm). Downgraded.
21. Publication bias test was not assessed due to the included trials were ≤10.
22. Data from RCTs begin with a grade of “HIGH”. Downgraded for imprecision.
23. The RoB for 1 of 4 trials were fair quality.
24. The number of included trials were less or equal to 5. Downgraded.
25. The number of participants was less than <400. Downgraded.
26. Publication bias test was not assessed due to the included trials were ≤10.
27. Data from RCTs begin with a grade of “HIGH”. Downgraded for RoB, indirectness and imprecision.
28. The RoB for 1 of 5 trials were fair and poor quality.
29. I^2^=70.2, P heterogeneity =0.009. The sensitivity analysis did not explain the source of heterogeneity. Downgraded.
30. The number of included trials were less or equal to 5. Downgraded.
31. The number of participants was less than <400. Downgraded.
32. Publication bias test was not assessed due to the included trials were ≤10.
33. Data from RCTs begin with a grade of “HIGH”. Downgraded for RoB, inconsistency, indirectness and imprecision.

**Supplementary Table 5.** Meta-analysis showing the effect of Probiotics/synbiotics supplementation on body weight (kilograms) based on several subgroups

|  |  | **Meta-analysis** | | **Heterogeneity** | | | |
| --- | --- | --- | --- | --- | --- | --- | --- |
| **Study group** | **Number of Studies** | **WMD (95%CI)** | **P effect** | **Q statistic** | **P within**  **group** | **I^2^ (%)** | **P between**  **group** |
| **Study location** |  |  |  |  |  |  |  |
| Asia | 23 | -0.40 (-0.71, -0.08) | 0.01 | 94.72 | <0.001 | 76.7 | 0.22 |
| Europe | 4 | -1.63 (-3.57, 0.31) | 0.10 | 27.66 | <0.001 | 89.2 |  |
| **Age** |  |  |  |  |  |  |  |
| < 60 | 23 | -0.54 (-0.92, -0.16) | 0.006 | 128.31 | <0.001 | 82.9 | 0.34 |
| ≥ 60 | 4 | -0.27 (-0.67, 0.13) | 0.19 | 0.15 | 0.98 | 0 |  |
| **Sex** |  |  |  |  |  |  |  |
| Male | 2 | -0.39 (-0.94, 0.16) | 0.16 | 0.67 | 0.41 | 0 | 0.74 |
| Female | 1 | -0.16 (-1.09, 0.77) | 0.74 | 0 | _ | _ |  |
| Both | 24 | -0.53 (-0.90, -0.17) | 0.004 | 127.78 | <0.001 | 82 |  |
| **Duration (week)** |  |  |  |  |  |  | 0.77 |
| < 12 | 14 | -0.46 (-0.86, -0.07) | 0.02 | 53.26 | <0.001 | 75.8 |  |
| ≥ 12 | 14 | -0.56 (-1.11, -0.01) | 0.046 | 74.90 | <0.001 | 82.6 |  |
| **Probiotic dose (CFU/d)** |  |  |  |  |  |  | 0.13 |
| ≤10×10^9^ | 8 | -0.18 (-0.74, 0.38) | 0.53 | 2.40 | 0.002 | 68.8 |  |
| >10×10^9^ | 12 | -0.58 (-0.96, -0.20) | 0.003 | 27.18 | 0.004 | 59.5 |  |
| **Probiotics/synbiotics type** |  |  |  |  |  |  | 0.32 |
| Synbiotics | 6 | -1.28 (-2.27, -0.29) | 0.01 | 59.08 | <0.001 | 91.5 |  |
| Bifidobacterium | 2 | 1.08 (-1.74, 3.9) | 0.45 | 7.13 | 0.008 | 86.5 |  |
| Lactobacillus | 9 | -0.31 (-0.71, 0.09) | 0.13 | 13.78 | 0.09 | 41.9 |  |
| Bifidobacterium & Lactobacillus | 8 | - 0.44 (-1.08, 0.21) | 0.18 | 40.53 | <0.001 | 85.2 |  |
| Mixed | 3 | -0.81 (-1.85, 0.23) | 0.13 | 4.07 | 0.13 | 50.9 |  |
| **BMI status** |  |  |  |  |  |  | 0.29 |
| Normal | 5 | -0.51 (-1.49, 0.47) | 0.31 | 26.38 | <0.001 | 84.8 |  |
| Overweight & Obese | 15 | -0.50 (-0.94, -0.07) | 0.02 | 56.74 | <0.001 | 75.3 |  |
| Mixed population | 7 | -0.38 (-1.14, 0.38) | 0.33 | 43.18 | <0.001 | 86.1 |  |
| **Study** **quality** |  |  |  |  |  |  | 0.24 |
| Good | 21 | -0.55 (-0.90, -0.20) | 0.002 | 93.43 | <0.001 | 78.6 |  |
| Fair | 4 | -0.07 (-0.51, 0.37) | 0.75 | 1.06 | 0.79 | 0 |  |
| Poor | 2 | 0.30 (-5.03, 5.09) | 0.99 | 21.18 | <0.001 | 95.3 |  |
| 1 dosage convert to logarithm*10^9^ | | | | | | | |

**Supplementary Table 6.** Meta-analysis showing the effect of Probiotics/synbiotics supplementation on BMI (Kg/m^2^) based on several subgroups

|  |  | **Meta-analysis** | | **Heterogeneity** | | | |
| --- | --- | --- | --- | --- | --- | --- | --- |
| **Study group** | **Number of Studies** | **WMD (95%CI)** | **P effect** | **Q statistic** | **P within**  **group** | **I^2^ (%)** | **P between**  **group** |
| **Study location** |  |  |  |  |  |  |  |
| Asia | 26 | -0.23 (-0.39, -0.07) | 0.005 | 190.84 | <0.001 | 86.9 | 0.64 |
| Europe | 4 | -0.33 (-0.72, 0.06) | 0.10 | 9.77 | 0.02 | 69.3 |  |
| **Age** |  |  |  |  |  |  |  |
| < 60 | 25 | -0.28 (-0.46, -0.10) | 0.002 | 199.78 | <0.001 | 88 | 0.03 |
| ≥ 60 | 5 | -0.04 (-0.17, 0.08) | 0.49 | 0.75 | 0.94 | 0 |  |
| **Sex** |  |  |  |  |  |  |  |
| Male | 2 | -0.16 (-0.36, 0.04) | 0.11 | 0.63 | 0.43 | 0 | 0.54 |
| Female | 1 | -0.05 (-0.44, 0.39) | 0.80 | 0 | _ | _ |  |
| Both | 27 | -0.26 (-0.42, -0.09) | 0.002 | 202.52 | <0.001 | 87.2 |  |
| **Duration (week)** |  |  |  |  |  |  | 0.17 |
| < 12 | 25 | -0.25 (-0.41, -0.08) | 0.004 | 185.88 | <0.001 | 87.1 |  |
| ≥ 12 | 5 | -0.20 (-0.53, 0.12) | 0.23 | 17.50 | 0.002 | 77.1 |  |
| **Probiotic dose (CFU/d)** |  |  |  |  |  |  | 0.38 |
| ≤10×10^9^ | 10 | -0.06 (-0.25, 0.12) | 0.51 | 26.50 | 0.002 | 66 |  |
| >10×10^9^ | 11 | -0.20 (-0.44, 0.03) | 0.09 | 56.28 | <0.001 | 82.2 |  |
| **Probiotics/synbiotics type** |  |  |  |  |  |  | 0.40 |
| Synbiotics | 7 | -0.24 (-0.45, -0.03) | 0.03 | 19.26 | 0.004 | 68.8 |  |
| Bifidobacterium | 2 | -1.04 (-3.10, 1.01) | 0.32 | 76.72 | <0.001 | 98.7 |  |
| Lactobacillus | 10 | -0.05 (-0.22, 0.11) | 0.58 | 20.00 | 0.02 | 55 |  |
| Bifidobacterium & Lactobacillus | 9 | -0.21 (-0.51, 0.10) | 0.18 | 70.77 | <0.001 | 88.7 |  |
| Saccharomyces | 1 | -0.4 (-0.78, -0.01) | 0.04 | 0.0 | - | - |  |
| Mixed | 3 | -0.28 (-0.52, -0.03) | 0.02 | 0.43 | 0.81 | 0.0 |  |
| **BMI status** |  |  |  |  |  |  | 0.29 |
| Normal | 6 | -0.08 (-0.24, 0.09) | 0.36 | 7.24 | 0.20 | 31 |  |
| Overweight & Obese | 18 | -0.16 (-0.28, -0.05) | 0.007 | 39.73 | 0.001 | 31 |  |
| Mixed population | 6 | -0.58 (-1.24, 0.07) | 0.08 | 148.52 | <0.001 | 96.6 |  |
| **Study quality** |  |  |  |  |  |  | 0.42 |
| Good | 20 | -0.15 (-0.30, -0.005) | 0.04 | 95.99 | <0.001 | 80.2 |  |
| Fair | 6 | -0.23 (-0.51, 0.04) | 0.09 | 14.18 | 0.01 | 64.7 |  |
| Poor | 4 | -0.24 (-0.76, 0.29) | 0.10 | 78.33 | <0.001 | 96.2 |  |

**Supplementary Table 7.** Meta-analysis showing the effect of Probiotics/synbiotics supplementation on waist circumference (cm) based on several subgroups

|  |  | **Meta-analysis** | | **Heterogeneity** | | | |
| --- | --- | --- | --- | --- | --- | --- | --- |
| **Study group** | **Number of Studies** | **WMD (95%CI)** | **P effect** | **Q statistic** | **P within**  **group** | **I^2^ (%)** | **P between**  **group** |
| **Study location** |  |  |  |  |  |  |  |
| Asia | 7 | -1.01 (-1.88, -0.14) | 0.02 | 2.10 | 0.95 | 0 | 0.78 |
| Europe | 4 | -0.88 (-1.31, -0.45) | <0.001 | 0.96 | 0.81 | 0 |  |
| **Age** |  |  |  |  |  |  |  |
| < 60 | 9 | -0.91 (-1.29, -0.52) | <0.001 | 2.21 | 0.99 | 0 | 0.84 |
| ≥ 60 | 2 | -0.51 (-4.42, 3.39) | 0.80 | 0.89 | 0.34 | - |  |
| **Sex** |  |  |  |  |  |  |  |
| Male | 2 | -0.44 (-3.77, 2.88) | 0.79 | 0.36 | 0.55 | 0 | 0.93 |
| Female | 2 | -0.65 (-2.76, 1.47) | 0.55 | 0.33 | 0.56 | 0 |  |
| Both | 8 | -0.92 (-1.31, -0.52) | <0.001 | 2.31 | 0.94 | 0 |  |
| **Duration (week)** |  |  |  |  |  |  | 0.95 |
| < 12 | 6 | -0.96 (-1.37, -0.55) | <0.001 | 1.81 | 0.87 | 0 |  |
| ≥ 12 | 5 | -0.91 (-2.73, 0.92) | 0.33 | 0.97 | 0.9 | 0 |  |
| **Probiotic dose (CFU/d)** |  |  |  |  |  |  | 0.72 |
| ≤10×10^9^ | 2 | -1.48 (-3.11, -0.16) | 0.08 | 1.04 | 0.31 | 3.5 |  |
| >10×10^9^ | 7 | -0.86 (-1.26, -0.46) | <0.001 | 0.59 | 0.99 | 0 |  |
| **Probiotics/synbiotics type** |  |  |  |  |  |  | 0.91 |
| Synbiotics | 1 | -3.00 (-9.48, 3.48) | 0.36 | 0 | _ | _ |  |
| Lactobacillus | 5 | -1.13 (-2.51, 0.24) | 0.10 | 2.50 | 0.64 | 0 |  |
| Bifidobacterium & Lactobacillus | 3 | -0.87 (-1.98, 0.25) | 0.12 | 0.05 | 0.82 | 0.0 |  |
| Mixed | 2 | -0.87 (-1.3, -0.44) | <0.001 | 0.05 | 0.81 | 0.0 |  |
| **BMI status** |  |  |  |  |  |  | 0.80 |
| Overweight & Obese | 7 | -0.92 (-1.33, -0.51) | <0.001 | 2.67 | 0.85 | 0 |  |
| Mixed population | 4 | -0.77 (-1.85, 0.30) | 0.16 | 0.41 | 0.98 | 0 |  |
| **Study quality** |  |  |  |  |  |  | 0.47 |
| Good | 8 | -0.92 (-1.31, -0.53) | <0.001 | 2.58 | 0.96 | 0 |  |
| Fair | 3 | 0.33 (-3.01, 3.67) | 0.85 | 0.03 | 0.99 | 0 |  |

| **Supplemental Table 8.** Meta-regression between changes in body composition indices and administered probiotics/synbiotics supplementation | | | |
| --- | --- | --- | --- |
|  | Slope | 95% CI | p-Value |
| **Body weight** |  |  |  |
| Mean age | 0.02 | (-0.04, 0.08) | 0.51 |
| Sample size | 0.03 | (0.00, 0.05) | 0.02 |
| Study duration | -0.07 | (-0.16, 0.01) | 0.08 |
| Probiotic dosage^1^ | -0.12 | (-0.35, 0.10) | 0.28 |
| **BMI** |  |  |  |
| Mean age | 0.00 | (-0.01, 0.02) | 0.73 |
| Sample size | -0.01 | (-0.02, 0.01) | 0.32 |
| Study duration | 0.01 | (-0.02, 0.04) | 0.41 |
| Probiotic dosage^1^ | -0.03 | (-0.14, 0.07) | 0.05 |
| **Waist circumference** |  |  |  |
| Mean age | 0.05 | (-0.07, 0.18) | 0.39 |
| Sample size | 0.03 | (-0.04, 0.10) | 0.41 |
| Study duration | 0.03 | (-0.15, 0.21) | 0.09 |
| Probiotic dosage^1^ | 0.02 | (-0.16, 0.21) | 0.83 |
| Dosage convert to logarithm*10^9^ | | | |

**
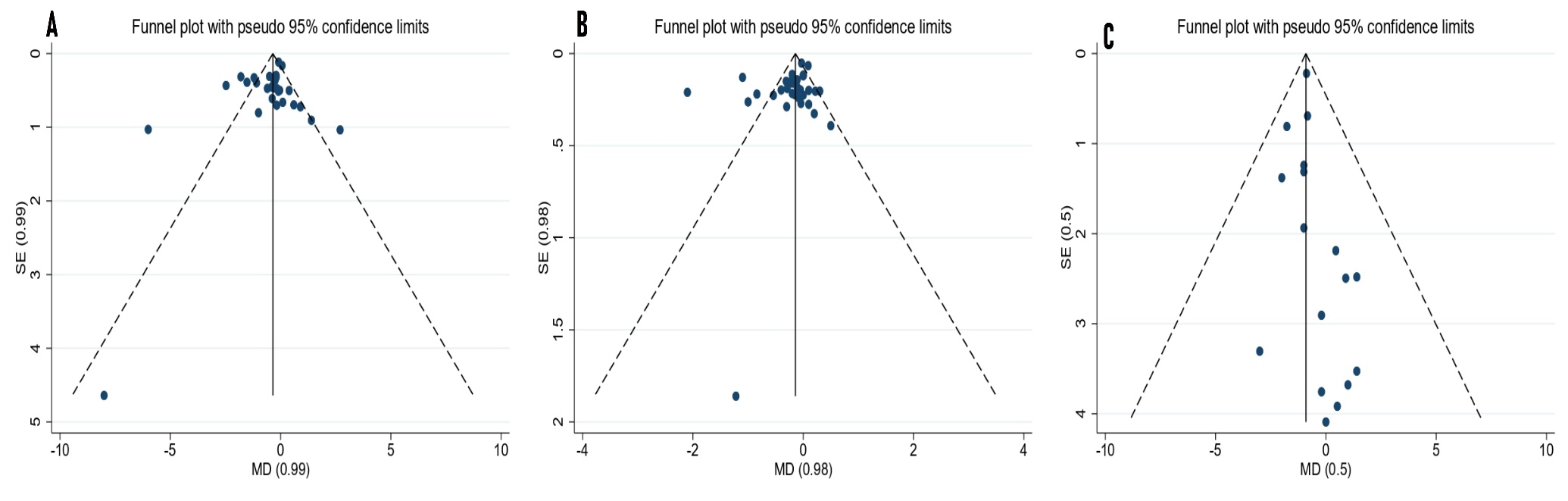
**

**Supplementary Figure 1.** Funnel plot the publication bias of randomized controlled trials for change of (A) body weight, (B) BMI, and (C) waist circumference following probiotic supplementation.

**References**

1. Mazloomi M, Tanideh N, Rezainzadeh A. THE EFFECTS SOYMILK FREMENTED WITH BIFIDOBACTERIUM LACTIS AND CONTAINING OMEGA-3 ON HAEMATOLOGICAL, OXIDATIVE STRESS, ANTI-OXIDANT AND INFLAMMATORY PARAMETERS IN TYPE 2 DIABETIC RATS. Iranian Journal of Diabetes and Metabolism. 2015;14(6):379-89.

2. Asemi Z, Alizadeh S-A, Ahmad K, Goli M, Esmaillzadeh A. Effects of beta-carotene fortified synbiotic food on metabolic control of patients with type 2 diabetes mellitus: a double-blind randomized cross-over controlled clinical trial. Clinical nutrition. 2016;35(4):819-25.

3. Kobyliak N, Abenavoli L, Falalyeyeva T, Mykhalchyshyn G, Boccuto L, Kyriienko D, et al. Beneficial effects of probiotic combination with omega-3 fatty acids in NAFLD: a randomized clinical study. 2018.

4. Kobyliak N, Falalyeyeva T, Mykhalchyshyn G, Molochek N, Savchuk O, Kyriienko D, et al. Probiotic and omega-3 polyunsaturated fatty acids supplementation reduces insulin resistance, improves glycemia and obesity parameters in individuals with type 2 diabetes: A randomised controlled trial. Obesity Medicine. 2020;19:100248.

5. Raygan F, Ostadmohammadi V, Asemi Z. The effects of probiotic and selenium co-supplementation on mental health parameters and metabolic profiles in type 2 diabetic patients with coronary heart disease: a randomized, double-blind, placebo-controlled trial. Clinical Nutrition. 2019;38(4):1594-8.

6. Ahmadian F, Ejtahed H, Javadi M, Razmpoosh E, Mirmiran P, Azizi F. The effects of probiotic supplementation on glycemic control, insulin resistance and inflammatory biomarkers of type 2 diabetic patients. 2017.

7. Firouzi S, Mohd-Yusof B-N, Majid H-A, Ismail A, Kamaruddin N-A. Effect of microbial cell preparation on renal profile and liver function among type 2 diabetics: a randomized controlled trial. BMC complementary and alternative medicine. 2015;15(1):1-10.

8. Kobyliak N, Abenavoli L, Mykhalchyshyn G, Kononenko L, Boccuto L, Kyriienko D, et al. A multi-strain probiotic reduces the fatty liver index, cytokines and aminotransferase levels in NAFLD patients: evidence from a randomized clinical trial. 2018.

9. Mobini R, Kovatcheva P, Tremaroli V, Karlsson F, Levin M, Ljungberg M, et al., editors. Metabolic effects of dietary supplementation with Lactobacillus reuteri DSM 17938: a randomised proof-of-concept study in type 2 diabetes. Diabetologia; 2015: SPRINGER 233 SPRING ST, NEW YORK, NY 10013 USA.

10. Birkeland E, Gharagozlian S, Birkeland KI, Valeur J, Måge I, Rud I, et al. Prebiotic effect of inulin-type fructans on faecal microbiota and short-chain fatty acids in type 2 diabetes: a randomised controlled trial. European journal of nutrition. 2020;59:3325-38.

11. Canfora EE, van der Beek CM, Hermes GD, Goossens GH, Jocken JW, Holst JJ, et al. Supplementation of diet with galacto-oligosaccharides increases bifidobacteria, but not insulin sensitivity, in obese prediabetic individuals. Gastroenterology. 2017;153(1):87-97. e3.

12. Dehghan P, Farhangi MA, Tavakoli F, Aliasgarzadeh A, Akbari AM. Impact of prebiotic supplementation on T-cell subsets and their related cytokines, anthropometric features and blood pressure in patients with type 2 diabetes mellitus: a randomized placebo-controlled trial. Complementary therapies in medicine. 2016;24:96-102.

13. Gonai M, Shigehisa A, Kigawa I, Kurasaki K, Chonan O, Matsuki T, et al. Galacto-oligosaccharides ameliorate dysbiotic Bifidobacteriaceae decline in Japanese patients with type 2 diabetes. Beneficial microbes. 2017;8(5):705-16.

14. Jafari T, Faghihimani E, Feizi A, Iraj B, Javanmard SH, Esmaillzadeh A, et al. Effects of vitamin D-fortified low fat yogurt on glycemic status, anthropometric indexes, inflammation, and bone turnover in diabetic postmenopausal women: A randomised controlled clinical trial. Clinical nutrition. 2016;35(1):67-76.

15. Kobyliak N, Abenavoli L, Falalyeyeva T, Kovalchuk O, Kyriienko D, Komisarenko I. Metabolic Benefits of Probiotic Combination with Absorbent Smectite in type 2 Diabetes Patients a Randomised Controlled Trial. Reviews on Recent Clinical Trials. 2021;16(1):109-19.

16. Kobyliak N, Abenavoli L, Mykhalchyshyn G, Falalyeyeva T, Tsyryuk O, Kononenko L, et al. Probiotics and smectite absorbent gel formulation reduce liver stiffness, transaminase and cytokine levels in NAFLD associated with type 2 diabetes: a randomized clinical study. Clinical Diabetology. 2019;8(4):205-14.

17. Król E, Krejpcio Z, Byks H, Bogdański P, Pupek-Musialik D. Effects of chromium brewer’s yeast supplementation on body mass, blood carbohydrates, and lipids and minerals in type 2 diabetic patients. Biological trace element research. 2011;143(2):726-37.

18. Lang R, Wang X-H, Li A-F, Liang Y, Zhu B-C, Shi B, et al. Effects of Jian Pi Qu Shi Formula on intestinal bacterial flora in patients with idiopathic membranous nephropathy: A prospective randomized controlled trial. Chronic Diseases and Translational Medicine. 2020;6(2):124-33.

19. Pino JL, Mujica V, Arredondo M. Effect of dietary supplementation with oat β-glucan for 3 months in subjects with type 2 diabetes: A randomized, double-blind, controlled clinical trial. Journal of Functional Foods. 2021;77:104311.

20. Urita Y, Noda T, Watanabe D, Iwashita S, Hamada K, Sugimoto M. Effects of a soybean nutrition bar on the postprandial blood glucose and lipid levels in patients with diabetes mellitus. International journal of food sciences and nutrition. 2012;63(8):921-9.

21. Abbasi B, Mirlohi M, Daniali M, Ghiasvand R. Effects of probiotic soymilk on lipid panel in type 2 diabetic patients with nephropathy: A double-blind randomized clinical trial. Prog Nutr. 2018;20:70-8.

22. Andreasen AS, Larsen N, Pedersen-Skovsgaard T, Berg RM, Møller K, Svendsen KD, et al. Effects of Lactobacillus acidophilus NCFM on insulin sensitivity and the systemic inflammatory response in human subjects. British journal of nutrition. 2010;104(12):1831-8.

23. Arani NM, Emam-Djomeh Z, Tavakolipour H, Sharafati-Chaleshtori R, Soleimani A, Asemi Z. The effects of probiotic honey consumption on metabolic status in patients with diabetic nephropathy: a randomized, double-blind, controlled trial. Probiotics and antimicrobial proteins. 2019;11(4):1195-201.

24. Bahijiri SM, Mira SA, Mufti AM, Ajabnoor MA. The effects of inorganic chromium and brewer's yeast supplementation on glucose tolerance, serum lipids and drug dosage in individuals with type 2 diabetes. Saudi medical journal. 2000;21(9):831-7.

25. Bahmani F, Tajadadi-Ebrahimi M, Kolahdooz F, Mazouchi M, Hadaegh H, Jamal A-S, et al. The consumption of synbiotic bread containing Lactobacillus sporogenes and inulin affects nitric oxide and malondialdehyde in patients with type 2 diabetes mellitus: randomized, double-blind, placebo-controlled trial. Journal of the American College of Nutrition. 2016;35(6):506-13.

26. Bayat A, Azizi-Soleiman F, Heidari-Beni M, Feizi A, Iraj B, Ghiasvand R, et al. Effect of cucurbita ficifolia and probiotic yogurt consumption on blood glucose, lipid profile, and inflammatory marker in type 2 diabetes. International Journal of Preventive Medicine. 2016;7.

27. Bazyar H, Maghsoumi-Norouzabad L, Yarahmadi M, Gholinezhad H, Moradi L, Salehi P, et al. The impacts of synbiotic supplementation on periodontal indices and biomarkers of oxidative stress in type 2 diabetes mellitus patients with chronic periodontitis under non-surgical periodontal therapy. A double-blind, placebo-controlled trial. Diabetes, metabolic syndrome and obesity: targets and therapy. 2020;13:19.

28. Dalal S, Nicolle L, Marrs CF, Zhang L, Harding G, Foxman B. Long-term Escherichia coli asymptomatic bacteriuria among women with diabetes mellitus. Clinical Infectious Diseases. 2009;49(4):491-7.

29. Ejtahed H, Mohtadi-Nia J, Homayouni-Rad A, Niafar M, Asghari-Jafarabadi M, Mofid V, et al. Effect of probiotic yogurt containing Lactobacillus acidophilus and Bifidobacterium lactis on lipid profile in individuals with type 2 diabetes mellitus. Journal of dairy science. 2011;94(7):3288-94.

30. Ejtahed HS, Mohtadi Nia J, Homayouni Rad A, Niafar M, Asghari Jafarabadi M, Mofid V. The effects of probiotic and conventional yoghurt on diabetes markers and insulin resistance in type 2 diabetic patients: a randomized controlled clinical trial. Iranian journal of endocrinology and metabolism. 2011;13(1):112.

31. Ejtahed HS, Mohtadi-Nia J, Homayouni-Rad A, Niafar M, Asghari-Jafarabadi M, Mofid V. Probiotic yogurt improves antioxidant status in type 2 diabetic patients. Nutrition. 2012;28(5):539-43.

32. Farrokhian A, Raygan F, Soltani A, Tajabadi-Ebrahimi M, Esfahani MS, Karami AA, et al. The effects of synbiotic supplementation on carotid intima-media thickness, biomarkers of inflammation, and oxidative stress in people with overweight, diabetes, and coronary heart disease: A randomized, double-blind, placebo-controlled trial. Probiotics and antimicrobial proteins. 2019;11(1):133-42.

33. Feizollahzadeh S, Ghiasvand R, Rezaei A, Khanahmad H, Hariri M. Effect of probiotic soy milk on serum levels of adiponectin, inflammatory mediators, lipid profile, and fasting blood glucose among patients with type II diabetes mellitus. Probiotics and antimicrobial proteins. 2017;9(1):41-7.

34. Ghafouri A, Zarrati M, Shidfar F, Heydari I, Shoormasti RS, Eslami O. Effect of synbiotic bread containing lactic acid on glycemic indicators, biomarkers of antioxidant status and inflammation in patients with type 2 diabetes: a randomized controlled trial. Diabetology & metabolic syndrome. 2019;11(1):1-9.

35. Hariri M, Salehi R, Feizi A, Mirlohi M, Ghiasvand R, Habibi N. A randomized, double-blind, placebo-controlled, clinical trial on probiotic soy milk and soy milk: effects on epigenetics and oxidative stress in patients with type II diabetes. Genes & nutrition. 2015;10(6):1-8.

36. Hsieh M-C, Tsai W-H, Jheng Y-P, Su S-L, Wang S-Y, Lin C-C, et al. The beneficial effects of Lactobacillus reuteri ADR-1 or ADR-3 consumption on type 2 diabetes mellitus: a randomized, double-blinded, placebo-controlled trial. Scientific reports. 2018;8(1):1-11.

37. Judiono J, Hadisaputro S, Indranila K, Cahyono B, Suzery M, Widiastuti Y, et al. Effects of clear kefir on biomolecular aspects of glycemic status of type 2 diabetes mellitus (T2DM) patients in Bandung, West Java [study on human blood glucose, c peptide and insulin]. Functional foods in health and disease. 2014;4(8):340-8.

38. Kassaian N, Feizi A, Aminorroaya A, Amini M. Probiotic and synbiotic supplementation could improve metabolic syndrome in prediabetic adults: A randomized controlled trial. Diabetes & Metabolic Syndrome: Clinical Research & Reviews. 2019;13(5):2991-6.

39. Kassaian N, Feizi A, Rostami S, Aminorroaya A, Yaran M, Amini M. The effects of 6 mo of supplementation with probiotics and synbiotics on gut microbiota in the adults with prediabetes: A double blind randomized clinical trial. Nutrition. 2020;79:110854.

40. KHOSRAVI‐BOROUJENI H, Rostami A, Ravanshad S, Esmaillzadeh A. Favorable effects on metabolic risk factors with daily brewer’s yeast in type 2 diabetic patients with hypercholesterolemia: A semi‐experimental study. Journal of diabetes. 2012;4(2):153-8.

41. Kooshki AA, Tofighiyan T, Rakhshani MH. Effects of synbiotics on inflammatory markers in patients with type 2 diabetes mellitus. Global journal of health science. 2015;7(7):1.

42. Mahboobi S, Iraj B, Maghsoudi Z, Feizi A, Ghiasvand R, Askari G, et al. The effects of probiotic supplementation on markers of blood lipids, and blood pressure in patients with prediabetes: a randomized clinical trial. International journal of preventive medicine. 2014;5(10):1239.

43. Mazloom Z, Yousefinejad A, Dabbaghmanesh MH. Effect of probiotics on lipid profile, glycemic control, insulin action, oxidative stress, and inflammatory markers in patients with type 2 diabetes: a clinical trial. Iranian journal of medical sciences. 2013;38(1):38.

44. Mirmiranpour H, Huseini HF, Derakhshanian H, Khodaii Z, Tavakoli-Far B. Effects of probiotic, cinnamon, and synbiotic supplementation on glycemic control and antioxidant status in people with type 2 diabetes; a randomized, double-blind, placebo-controlled study. Journal of Diabetes & Metabolic Disorders. 2020;19(1):53-60.

45. Moroti C, Magri LFS, de Rezende Costa M, Cavallini DC, Sivieri K. Effect of the consumption of a new symbiotic shake on glycemia and cholesterol levels in elderly people with type 2 diabetes mellitus. Lipids in health and disease. 2012;11(1):1-8.

46. Raygan F, Rezavandi Z, Bahmani F, Ostadmohammadi V, Mansournia MA, Tajabadi-Ebrahimi M, et al. The effects of probiotic supplementation on metabolic status in type 2 diabetic patients with coronary heart disease. Diabetology & metabolic syndrome. 2018;10(1):1-7.

47. Rezaei M, Sanagoo A, Jouybari L, Behnampoo N, Kavosi A. The effect of probiotic yogurt on blood glucose and cardiovascular biomarkers in patients with type II diabetes: a randomized controlled trial. Evidence Based Care. 2017;6(4):26-35.

48. Sabatini S, Lauritano D, Candotto V, Silvestre F, Nardi G. Oral probiotics in the management of gingivitis in diabetic patients: a double blinded randomized controlled study. J Biol Regul Homeost Agents. 2017;31(2 Suppl 1):197-202.

49. Shakeri H, Hadaegh H, Abedi F, Tajabadi‐Ebrahimi M, Mazroii N, Ghandi Y, et al. Consumption of synbiotic bread decreases triacylglycerol and VLDL levels while increasing HDL levels in serum from patients with type‐2 diabetes. Lipids. 2014;49(7):695-701.

50. Simon M-C, Strassburger K, Nowotny B, Kolb H, Nowotny P, Burkart V, et al. Intake of Lactobacillus reuteri improves incretin and insulin secretion in glucose-tolerant humans: a proof of concept. Diabetes care. 2015;38(10):1827-34.

51. Soleimani A, Mojarrad MZ, Bahmani F, Taghizadeh M, Ramezani M, Tajabadi-Ebrahimi M, et al. Probiotic supplementation in diabetic hemodialysis patients has beneficial metabolic effects. Kidney international. 2017;91(2):435-42.

52. Soleimani A, Motamedzadeh A, Mojarrad MZ, Bahmani F, Amirani E, Ostadmohammadi V, et al. The effects of synbiotic supplementation on metabolic status in diabetic patients undergoing hemodialysis: a randomized, double-blinded, placebo-controlled trial. Probiotics and antimicrobial proteins. 2019;11(4):1248-56.

53. Tazakori Z, Zare M, Jafarabadi MA. Probiotic yogurt effect on macronutrients ingredients, blood glucose and lipid profile in type 2 diabetes. J Pak Med Assoc. 2017;67(7):1123.

54. Tonucci LB, Dos Santos KMO, de Oliveira LL, Ribeiro SMR, Martino HSD. Clinical application of probiotics in type 2 diabetes mellitus: A randomized, double-blind, placebo-controlled study. Clinical nutrition. 2017;36(1):85-92.

55. Venkataraman R, Jose P, Jose J. Impact of probiotics on health-related quality of life in Type II diabetes mellitus: a randomized single-blind, placebo-controlled study. Journal of Natural Science, Biology and Medicine. 2019;10(1):2.

56. Zhang Y, Gu Y, Ren H, Wang S, Zhong H, Zhao X, et al. Gut microbiome-related effects of berberine and probiotics on type 2 diabetes (the PREMOTE study). Nature communications. 2020;11(1):1-12.

57. Kushugulova A, Benberin V, Karabayeva R, Saduakhasova S, Kozhakhmetov S, Shakhabayeva G, et al. Randomized Clinical Trial: Efficacy of a New Synbiotic in Adults with Metabolic Syndrome. Central Asian journal of global health. 2013;2.

58. Parastouei K, Saeidipoor S, Sepandi M, Abbaszadeh S, Taghdir M. Effects of synbiotic supplementation on the components of metabolic syndrome in military personnel: a double-blind randomised controlled trial. BMJ Mil Health. 2020.

59. Alihosseini N, Moahboob S, Farrin N, Mobasseri M, Taghizadeh A, Ostadrahimi A. Effect of probiotic fermented milk (kefir) on serum level of insulin and homocysteine in type 2 diabetes patients. Acta Endocrinologica (Bucharest). 2017;13(4):431.

60. Ostadrahimi A, Taghizadeh A, Mobasseri M, Farrin N, Payahoo L, Gheshlaghi ZB, et al. Effect of probiotic fermented milk (kefir) on glycemic control and lipid profile in type 2 diabetic patients: a randomized double-blind placebo-controlled clinical trial. Iranian journal of public health. 2015;44(2):228.

61. Abbasi B, Ghiasvand R, Mirlohi M. Kidney function improvement by soy milk containing Lactobacillus plantarum A7 in type 2 diabetic patients with nephropathy: a double-blinded randomized controlled trial. Iranian journal of kidney diseases. 2017;11(1):36.

62. Asemi Z, Aarabi MH, Hajijafari M, Alizadeh S-A, Razzaghi R, Mazoochi M, et al. Effects of synbiotic food consumption on serum minerals, liver enzymes, and blood pressure in patients with type 2 diabetes: a double-blind randomized cross-over controlled clinical trial. International journal of preventive medicine. 2017;8.

63. Asemi Z, Zare Z, Shakeri H, Sabihi S-s, Esmaillzadeh A. Effect of multispecies probiotic supplements on metabolic profiles, hs-CRP, and oxidative stress in patients with type 2 diabetes. Annals of nutrition and metabolism. 2013;63(1-2):1-9.

64. Hariri M, Salehi R, Feizi A, Mirlohi M, Kamali S, Ghiasvand R. The effect of probiotic soy milk and soy milk on anthropometric measures and blood pressure in patients with type II diabetes mellitus: A randomized double-blind clinical trial. ARYA atherosclerosis. 2015;11(Suppl 1):74.

65. Kassaian N, Feizi A, Aminorroaya A, Jafari P, Ebrahimi MT, Amini M. The effects of probiotics and synbiotic supplementation on glucose and insulin metabolism in adults with prediabetes: a double-blind randomized clinical trial. Acta diabetologica. 2018;55(10):1019-28.

66. Miraghajani M, Zaghian N, Mirlohi M, Feizi A, Ghiasvand R. The impact of probiotic soy milk consumption on oxidative stress among type 2 diabetic kidney disease patients: a randomized controlled clinical trial. Journal of Renal Nutrition. 2017;27(5):317-24.

67. Mohamadshahi M, Veissi M, Haidari F, Javid AZ, Mohammadi F, Shirbeigi E. Effects of probiotic yogurt consumption on lipid profile in type 2 diabetic patients: A randomized controlled clinical trial. Journal of research in medical sciences: the official journal of Isfahan University of Medical Sciences. 2014;19(6):531.

68. Mykhal'chyshyn H, Bodnar P, Kobyliak N. Effect of probiotics on proinflammatory cytokines level in patients with type 2 diabetes and nonalcoholic fatty liver disease. Likars' ka sprava. 2013(2):56-62.

69. Yuan T, Zhao W, Cao Y, Li Q, Yao M, Hao X, et al. An efficacy and safety study of bifidobacterium tetragenous viable bacteria tablets in the treatment of constipation in patients with type 2 diabetes mellitus. Zhonghua nei ke za zhi. 2018;57(4):252-7.

70. Yuan T, Zhao W, Cao Y, Qi L, Yao M, Hao X, et al. Effect of Bifidobacterium tetragenous viable bacteria tablets on blood glucose level in patients with type 2 diabetes mellitus. Chinese Journal of Clinical Nutrition. 2017;25(4):205-13.
